# Evaluating sustainability: a retrospective cohort analysis of the Oxfordshire therapeutic community

**DOI:** 10.1186/s12888-016-0994-3

**Published:** 2016-08-11

**Authors:** Daniel Maughan, Rob Lillywhite, Steve Pearce, Toby Pillinger, Scott Weich

**Affiliations:** 1 Oxford Health NHS Foundation Trust, Warneford Hospital, Warneford Lane, Oxford, OX3 7JX UK; 2 Warwick Medical School, Gibbet Hill Campus, Coventry, CV4 7AL UK; 3 Life Sciences, Warwick University, Gibbet Hill Campus, Coventry, CV4 7AL UK; 4 South London and Maudsley NHS Foundation Trust, Bethlem Royal Hospital, Monks Orchard Road, Beckenham, BR3 3BX UK

**Keywords:** Sustainability, Personality disorders, Carbon footprint, Economic evaluation

## Abstract

**Background:**

Therapeutic communities (TCs) could reduce the health care use of people with personality disorder (Davies S, Campling P and Ryan K, Psychiatrist 23:79–83, 1999; Barr W, Kirkcaldy A, Horne A, Hodge S, Hellin K and Göpfert M, J Ment Health 19:412–421, 2010) and in turn reduce the financial and environmental costs of services. Our hypothesis is that 3 years following entry to a TC service, patients have reduced subsequent health care use and associated reductions in financial costs and carbon footprint.

**Methods:**

A retrospective 4-year cohort study examined changes in health care use following entry to the Oxfordshire TC service. Comparative analysis was undertaken on a treated (*n* = 40) and a control group (referred but who declined treatment; *n* = 45). Financial costs and carbon footprint of health care use were calculated using national tariffs and standard carbon conversion factors. Mean changes in these outcomes were compared over 1, 2 and 3 years and adjusted for costs and carbon footprints in the year prior to joining the TC service.

**Results:**

Compared to baseline, the group receiving TC care had greater reductions in financial costs and carbon footprint associated with A&E attendances (*p* = 0.04) and crisis mental health appointments (*p* = 0.04) than the control group. There were significantly greater reductions in carbon footprint for all secondary health care use, both physical and mental health care, after 3 years (*p* = 0.04) in the TC group.

**Conclusions:**

TC services may have the potential to reduce the financial cost and carbon footprint of health care.

## Background

The NHS has signed up to meet the Climate Change Act targets [1], which demand carbon emission reductions of 80 % by 2050. This statute was enacted because of the alarming and pressing issue of climate change. The provision of healthcare is known to be a major emitter of greenhouse gases (GHGs); in the UK, the NHS contributes 3 % of GHG emissions, while the US health system contributes about 8 % [1, 2]. The majority of GHG emissions associated with health care are likely due to clinical factors such as medication and clinical equipment, as opposed to buildings energy use [3]. The targets established by the Climate Change Act are very ambitious and it is likely that incremental improvements in healthcare delivery will not be enough to bring about the required reduction in GHG emissions. New models of care are required that are both cost effective and carbon efficient [4]. Since development of care models are rarely influenced by their potential environmental impact, new approaches are required that assess them on both the quality and outcome of care and their emission of GHGs. To inform this discussion, this paper explores the novel concept of evaluating the carbon footprint of care pathways in mental health through an analysis of the financial costs and GHG emissions associated with a therapeutic community (TC) service for those with personality disorder (PD). A TC is a form of psychosocial treatment based on a collaborative approach; particular emphasis is placed on empowerment, personal responsibility, shared decision making, and participation in communal activity.

Personality disorders are common conditions, affecting between 5 and 13 % of people living in the community and over 40 % of those seen in psychiatric outpatient departments [5]. People with PD present to services with a wide range of physical, mental and social problems such as self-harm, substance misuse, depression and suicide, housing problems and long-standing interpersonal difficulties [6]. Recent decades have witnessed growing concern about the effectiveness of treatments for people with PD [7, 8]. As a result, 11 pilot projects were set up across England between 2004 and 2007 to provide care for people with PD [6]. These provided varying models of therapy programmes. In 2013–14 the funding for these programmes changed from national to local commissioners, with subsequent restrictions on budgets. This study examines the TC intervention delivered by one of these pilot projects, based in Oxfordshire.

While there is evidence to suggest that TCs are clinically effective [9, 10], there remain uncertainties as to whether they are cost-effective [11]. In 2003, NICE stated that additional cost benefits would accrue following TC treatment, through reduced health care use such as A&E services, fewer mental health admissions and a reduction in the number of prescribed medications [8]. This finding is supported by a number of studies. Dolan (1996) found that costs were recovered within 2 years of entry to an inpatient TC service for PD due to reduced subsequent psychiatric care and prison use [12]. Chiesa (1996) also measured subsequent health care use following treatment in an inpatient psychotherapy service for PD and found a significant reduction in the use of physical and mental health services in the year following treatment [13]. There was also reduced psychotropic medication prescription, cigarette use and alcohol consumption, while employment rate increased [13]. Davies (1999) found that costs of a residential TC were recovered after 4 years due to reduced frequency and duration of psychiatric admissions alone [14]. Bateman (2003) examined day treatment for borderline personality disorder and found it was associated with reductions in subsequent health care use [15]. More recently, a study looking at a weekly psychotherapy service for people with PD found that costs could be recovered after 3 years [16]. Therefore, despite being costly, TCs for those with PD can provide wider financial savings for the health care system through reducing subsequent health care usage.

These clinical and economic evaluations did not consider the environmental consequences of TC treatment. Furthermore, we are not aware of any studies to date that have investigated the environmental costs of a secondary mental health service [17]. Importantly, recent methodological advances in carbon metrics have estimated the carbon footprint of health care according to type of clinical activity (e.g. primary care appointment or mental health admission), which should allow the reliable estimation of the carbon footprint of health care use across different services [3].

The aim of this study was to therefore determine how one service, the Oxfordshire Complex Needs Service, compares to treatment as usual, with regards to health care use and the subsequent financial costs and carbon footprint. Given the evidence that TCs can reduce future health care use, the addition of a TC service might also reduce the overall carbon footprint of the health care system. Our hypothesis is that 3 years following entry to a TC service, patients have reduced subsequent health care use and associated reductions in financial costs and carbon footprint.

## Method

### Design

A retrospective cohort study was undertaken to compare two groups over 4 years; those who attended the Oxfordshire TC service and those who were referred but declined further care.

Data on the TC group were obtained for the period covering 1 year prior to referral to 3 years after entry to TC. Twelve-month time periods were used in this study to analyse health care use before and after entry to TC. Corresponding data for the control group were collected over the same 4-year period (April 2010–April 2014).

Health care use was ascertained separately for primary care, secondary mental health care and secondary physical health care. For secondary mental and physical health care, data were obtained from electronic data records held by Information Services departments in the main local provider organisations (Oxford Health NHS Foundation Trust and Oxford University Hospitals Trust, respectively). Data was collected on: outpatient consultations, inpatient admissions, mental health crisis appointments and A&E attendances. No health use data from other providers was available and it is possible that some people received care elsewhere. Primary care data (on prescriptions and GP consultations) were only available for a minority of patients (~25 %) due to difficulties accessing data from primary care practices. Because of the high level of missing data from primary care, these were not included in further analyses.

Records were sourced using health care record numbers and all patient identifiers were removed prior to analysis.

### Setting

The Oxford TC service is delivered at four therapy centres that provide care of varying intensity. Patients were offered treatment over 18 months at one of the following locations: Oxford (3 to 5 days per week), Banbury or Wallingford (2 days per week), or Witney (1 day per week). All centres offered the same types of group interventions. All centres were analysed together as one sample.

### Participants

The TC group consisted of all those who had been referred to the service and had started treatment between April 1^st^ 2011 and 1^st^ April 2012. Patients in the control group were those who were referred during the same year (with a likely diagnosis of Personality Disorder) but did not attend their initial appointment and were subsequently discharged. During this year there were 188 patients who were discharged due to non-attendance. From this group, 45 were randomly chosen using a random number generator, as this represented a similar number to the TC group. The control group received treatment as usual, which might have included either primary care input or secondary mental health care or both.

### Measures

The financial costs associated with secondary health care use (other than the TC service) were calculated using national standard costs for the different clinical activities (see Table 1). It was assumed that the control group were not accessing any treatment (other than TAU) at this time.

**Table 1:** Financial costs and carbon conversion factors for health care use and other assumptions made

COST (£)	
A&E attendance	£113 ^b^
OPD appointment	£135 ^b^
Physical health cost per bed-day	£399 ^b^
Mental health OPD appointment	£100 ^b^
Mental health cost per bed-day	£430 ^b^
GP consultation	£45 ^b^
Bus journey	£2-assumed
Petrol	£1.40 / L-assumed
Cost for travel incl staff and patient travel (from survey)	£8.10/pt/wk (urban) £3.70/pt/wk (rural)
Cost of energy	£15.60 / kWh (British Gas standard price)
CARBON FOOTPRINT (kg CO_2_e)	
ED attendance / one bed day	91 kgCO_2_e ^c^
OPD appointment	56 kgCO_2_e ^c^
Mental health OPD appointment	59 kgCO_2_e ^c^
Mental health cost per bed day for INPT	97 kgCO_2_e ^c^
GP consultation	66 kgCO_2_e ^c^
Medication conversion factor	0.4 kgCO_2_e/£ ^a^
Energy conversion factor	0.5 kgCO_2_e / kWh ^a^
Bus conversion factor	0.1 kgCO_2_e / mile ^a^
Medium sized car conversion factor	0.2 kgCO_2_e / mile ^a^
Carbon footprint for travel incl staff and patient travel (from survey)	9 kgCO_2_e/pt/wk (urban) 7 kgCO_2_e /pt/wk (rural)
OTHER CONVERSION FACTORS OR ASSUMPTIONS MADE	
Average fuel consumption for car	60mpg
Type of car used	Small average car size
Total area of all treatment rooms and office space used by TC	185 m^2^
Carbon footprint per square metre of outpatient room	147kWh/m^2^ annually
Duration of equipment used	5 years
Cost of laptop / easy chair / desk chair / desk	£400 / £200 / £400 / £150
Salaries of staff	Taken as average salaries ^b^
Staff time dedicated to therapeutic community intervention and number of patients in service at any given time	Estimated by director of service

^a^ DEFRA [18]

^b^ Curtis Report 2013 [25]

^c^ SDU [3]

The carbon footprint of the TC intervention was estimated using a ‘bottom-up’ approach that used direct measurements of resource use in the TC service and established carbon conversion factors (see Table 1) [18] either to financial cost (procurement and energy use) or direct resource use (travel). Staff and patient travel for the TC service was measured using surveys, these included data on method of travel and postcodes of origin and destination. All staff employed by the TC service were surveyed, while patient travel data was obtained from a local survey based on 200 mental health patients in the same geographical area and organisation as the TC (details available from the authors on request). The footprints of each different resource type were summed to produce a total carbon footprint for the TC intervention.

The carbon footprints of all other clinical activities were obtained from a report published by the NHS Sustainable Development Unit [3] (see Table 1). This used a top-down approach that estimates the carbon footprint based on financial spend. This ‘top-down’ method was chosen as it was not feasible to directly measure all resource use involved in the delivery of secondary physical and mental health care. Instead the carbon footprint of a clinical activity is calculated by applying a carbon conversion factor to the financial cost of each resource category in the organisation (e.g. pharmaceuticals, fuel, equipment etc.). Carbon conversion factors are determined from a widely accepted economic modeling technique termed multi-region-input-output analysis [19]. Carbon footprints were derived for each category at an organisational level and then scaled down to provide carbon footprints for individual clinical activities, according to the number of clinical activities occurring in the organisation [20]. Travel associated with other secondary health care use was not included due to a lack of available data.

### Statistical analysis

The main outcome was assessed at 3 years following entry to the TC service. Summary statistics were used to understand the main effects. Differences were assumed to be significant at a two-tailed 5 % level. Mean costs, both financial and environmental, were compared between groups using t-tests; change scores (post annual average minus baseline year) were used for this analysis. Percentile bootstrapped 95 % confidence interval and corresponding *p*-values are presented to account for non-normality of data. All analyses were carried out in Stata SE 13 (Stata Corp 2013)

## Results

The TC group contained 40 patients (females = 29, males = 11). Numbers from each therapy centre were as follows: Oxford (*n* = 12), Banbury (*n* = 11), Wallingford (*n* = 10), and Witney (*n* = 7). The control group contained 45 patients (females = 33, males = 12). The mean age was 34 years for the TC group and 39 years for the control group. In the treatment group, 20 stayed the full 18-month course, while the minimum duration of treatment was 2 months (*n* = 4). Due to the retrospective nature of this study we were unable to collect other data regarding patient characteristics. Mean duration in treatment was 12.9 months (SD = 6.4); median duration was 18 months. Table 2 shows the financial cost and carbon footprint of the TC intervention. Most of the financial costs are made up of staff costs, while the carbon footprint is mostly contributed by travel, followed by energy use.

**Table 2:** Mean financial cost and carbon footprint of the TC intervention per patient

Costs for one course of TC	Financial (£)	Carbon footprint (kgCO_2_e)
Travel	282	386
Staff pay	4559	n/a
Energy use	77	239
Procurement	63	14
Total	4981	639

At 1 year following entry to TC, the TC group had significantly lower financial cost and carbon footprint associated with reduced A&E attendances and crisis appointments compared to control (Tables 3 and 4). At 2 years following entry, the TC group had significantly lower financial cost and carbon footprint of physical health inpatient days and crisis appointments compared to the control. While the TC intervention is costly in both financial and carbon terms, these costs are increasingly offset over the 3 years by reductions in health care use. This is demonstrated by the statistically significant net carbon footprint reduction at 3 years for all secondary health care, compared to controls, after taking the TC costs into account. (MD = 802kgCO_2_e, *p* = 0.04 95 % CI = 54–1068). The average annual cost for TC appears to decrease as the time period increases as the costs are averaged across each time period (Tables 3 and 4).

**Table 3:** Financial cost savings for secondary care for the TC group per patient per year, adjusted for baseline year and controls

Type of secondary care	Variable	Mean cost difference between groups adjusted for costs in baseline year (95 % CI); *P* value		
		Over 1 year	Over 2 years	Over 3 years
Physical health care	Secondary care appt (physical)	£7 (−176, 190) *p* = 0.94	£4 (−148, 155) *p* = 0.96	−£23 (−177, 132) *p* = 0.77
	Physical inpt days	£370 (−6, 746) *p* = 0.05	£255 (21, 489) *p* = 0.03*	£536 (−157, 1229) *p* = 0.13
	A&E attendance	£58 (4, 112) *p* = 0.04*	£39 (−13, 91) *p* = 0.14	£40 (−11, 90) *p* = 0.12
Mental health care	Secondary care appt (mental)	£506 (−9, 1020) *p* = 0.05	£398 (−33, 830) *p* = 0.07	£394 (−41, 829) *p* = 0.08
	Crisis appts	£141 (5, 276) *p* = 0.04*	£103 (10, 197) *p* = 0.03*	£103 (−11, 218) *p* = 0.08
	Mental inpt days	£1566 (−821, 3953) *p* = 0.20	£1294 (−1155, 3742) *p* = 0.30	£1613 (−861, 4087) *p* = 0.20
	TC service	−£4981	−£2491	−£1,660
Total secondary health care		−£2333 *p* = 0.06	−£398 *p* = 0.14	£1003 *p* = 0.07

Positive values indicate savings in favour of the TC group

* significant at 5 % level

**Table 4:** Carbon footprint reductions for secondary care for the TC group per patient per year, adjusted for baseline year and controls

Type of secondary care	Variable	Mean carbon footprint difference between groups adjusted for carbon footprint in baseline year (95 % CI); *P* value		
		Over 1 year	Over 2 years	Over 3 years
Physical health care	Secondary care appt (physical)	3 kgCO_2_e (−73, 79) *p* = 0.94	2 kgCO_2_e (−61, 64) *p* = 0.96	−9 kgCO_2_e (−73, 55) *p* = 0.77
	Physical inpt days	84 kgCO_2_e (−1, 170) *p* = 0.05	58 kgCO_2_e (5, 112) *p* = 0.03*	122 kgCO_2_e (−36, 280) *p* = 0.13
	A&E attendance	47 kgCO_2_e (3, 90) *p* = 0.04*	32 kgCO_2_e (−10, 74) *p* = 0.14	32 kgCO_2_e (−9, 72) *p* = 0.12
Mental health care	Secondary care appt (mental)	298 kgCO_2_e (−5, 602) *p* = 0.05	235 kgCO_2_e (−19, 489) *p* = 0.07	233 kgCO_2_e (−24, 489) *p* = 0.08
	Crisis appts	83 kgCO_2_e (3, 163) *p* = 0.04*	61 kgCO_2_e (6, 116) *p* = 0.03*	61 kgCO_2_e (−7, 128) *p* = 0.08
	Mental inpt days	353 kgCO_2_e (−185, 892) *p* = 0.20	292 kgCO_2_e (−260, 844) *p* = 0.30	364 kgCO_2_e (−194, 922) *p* = 0.20
	TC service	−639 kgCO_2_e	−320 kgCO_2_e	−213 kgCO_2_e
Total secondary health care		229 kgCO_2_e *p* = 0.03*	359 kgCO_2_e *p* = 0.08	590 kgCO_2_e *p* = 0.04*

Positive values indicate savings in favour of the TC group

* significant at 5 % level

**Fig. 1:**
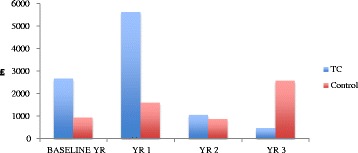
Average financial cost of secondary health care use per patient per year. Description of data: Financial outcomes of study

**Fig. 2:**
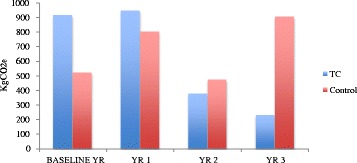
Average carbon footprint of secondary health care use per patient per year. Description of data: Carbon footprint outcomes of study

Figures 1 and 2 show the financial costs and carbon footprint for secondary health care use per patient per year respectively. The costs and carbon footprint of the TC intervention are included in year 1. The baseline financial costs and carbon footprint for the two groups were significantly different. The control group was associated with higher costs as a result of a larger number of mental health admissions and appointments. Apart from year 1, which for the TC group included the TC intervention, there is a pattern of escalating financial costs and carbon footprint in the control group and decreasing costs and carbon footprint in the TC group.

**Fig. 3:**
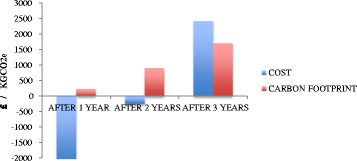
Total financial cost and carbon footprint savings for secondary care for the TC group per patient per year compared with control group and adjusted for baseline year. Description of data: Total outcomes of study adjusted for controls

Within 1 year of the program starting, the carbon footprint of TC is offset through reduced subsequent secondary health care use (Fig. 3). However, it takes until year 2 for the financial costs are recovered. By year 3, there are consistent savings in both financial cost and carbon footprint per patient, although only the carbon footprint reduction is statistically significant (£2409 and 1705 kgCO_2_e).

## Discussion

The aim of this study was to explore whether community based therapeutic interventions for personality disorder can deliver both cost savings and a smaller carbon footprint. This was a retrospective analysis based on an existing programme of care and therefore has a number of limitations.

The findings show, over a period of 3 years following admission to the TC service, patients have reduced health care use and associated reductions in carbon footprint. The financial savings were not significant but those for carbon footprint were. Given the median length of stay in TC was 18 months (and mean was 12.9 months), it is reasonable to assume that most patients in the treatment group were given an adequate course of therapy to realise therapeutic benefits.

These results suggest that the addition of a therapeutic community intervention to the local health care provision can reduce the carbon footprint of the wider physical and mental health care system. Despite the intensive nature of the TC intervention, given its long duration and high frequency of contact, it is unlikely to have a large carbon footprint. Verbal therapies do not require complex or expensive equipment to deliver the intervention, nor do they use carbon intensive medications [21]. Further, the provision of locally based therapy services in this model of TC minimises the costs and carbon footprint associated with patient travel.

To provide some reference for the scale of changes this study has shown, we display the overall differences against the average cost and carbon footprint of health care use per person in the UK. The budget for the NHS in England for 2015 was £116.4 billion, the NHS carbon footprint in 2015 was 22.8MtCO_2_e and the population of UK was 64.8 million in 2015. Therefore, assuming all health care used in the UK is from the NHS, the average cost of health care per person is £1800 and the average carbon footprint of health care per person is 351kgCO_2_e. Comparing these data to the results finds that the TC group’s average health care costs go from being on average £871 more expensive per year to £1312 less expensive than the average cost of health care in the UK per person. The control groups average costs increase from below the average health care costs per person in the UK to £779 more than the average UK cost. The average carbon footprint for the TC group’s health care use goes from being on average 565kgCO_2_e more per year to 117kgCO_2_e less than the average carbon footprint of health care in the UK per person. The control group’s carbon footprint increases from 172kgCO_2_e above the average carbon footprint of health care in the UK per person to being 555kgCO_2_e above the average.

While the cost and carbon footprint of admissions is the largest compared with other activities, this value is unlikely to vary much given that there are standard requirements for ward-based care. The assumptions that likely have the largest impact on the cost and carbon footprint of care delivered are those related to the TC intervention. The cost and carbon footprint of travel and the energy usage have the potential to vary considerably depending on what premises and energy supplier are used and whether the service is based in an urban or rural area. Given this service was based in both rural and urban areas and that the energy supplier provided a standard package, rather than from renewables, there is no reason to assume that this analysis was vulnerable to being skewed in either direction by these potential uncertainties.

### Strengths and limitations

This study is the first of its kind to measure the carbon footprint of a mental health service. It is an exploratory study that analyses the carbon footprint associated with a health care service using existing methodologies. However, as the study is observational and retrospective, there has been no randomisation between groups. As with all observational research, confounding by indication is difficult to exclude. In this study, the control group were patients who were referred to the TC service but did not attend appointments. This group is therefore possibly different to the TC group in any number of ways; but any differences were not explored in the course of the study. This may be illustrated by considering the higher health care use in the TC group in the baseline year, (see Figs. 1 and 2). Consequently, the financial costs and carbon footprint of the TC group may be over-estimated, as the health care use of these patients may have been easier to detect, conversely, while there may be missed information about either group, the control group (as non-attenders) may not have been as visible. We controlled for differences in prior health care service use by adjusting for health care use in the baseline year when comparing groups. Missing primary care data is also a concern, which led to excluding this data from the analysis. The control patients may have had higher primary care use relative to the TC group, but this could not be evaluated and we have had to assume this does not affect the results.

The expectation is that the requirement for some types of health care use, e.g. psychotropic medications, A&E attendances and crisis appointments will reduce following the TC intervention. The results confirm this to be true and a significant reduction was observed. In contrast, those types of health care that would not be expected to change following the intervention, e.g. physical health secondary care referrals, did not demonstrate significant reductions.

Estimating the carbon footprint of health care is problematic [3] and the top-down approaches used in this paper cannot fully describe the actual variation that exists in health care delivery. For example, one doctor might use less radiology or pathology tests than another. While the approach provides a pragmatic method for estimating the carbon footprint of clinical care, the assumption that each admission or appointment has a uniform cost is probably not true in practice. The cost or burden of infrastructure is mostly fixed and varies by occupancy, e.g. greater occupancy might lead to a reduced overall carbon footprint per patient. This use of typical or standardised carbon footprints per clinical activity is the main limitation with such top-down methods. However, as the carbon footprint of both the control and TC groups were estimated using this method, the relative difference is of almost as much value as the absolute difference and there is no reason to believe this limitation would affect the overall interpretation of the results [22].

The use of primary data in the estimation of the carbon footprints can also create issues (see Table 2). The biggest is likely to be ensuring that the inventory that supports the calculations is complete. This is an issue with all carbon footprint and life cycle assessment estimations since it is difficult to identify all activity data, e.g. overheads and managerial costs, that are associated the intervention [23]. As a consequence, all estimations require a number of assumptions to fill in missing data gaps; in this study, those assumptions include a proportion of staff time, equipment use and energy consumed by the TC group. In order to try to address overhead costs, reference costs used for staff salaries were those that included all overhead costs. This study only considered activity data associated with running the TC service. It did not consider the costs, either financial or carbon, of establishing the service. Further, this study only considered the environmental costs of treatment; it is likely that, as patients recover, the environmental costs of their lifestyle may change. Some may start driving to work again, others may decide to fly to Australia to celebrate their recovery; a highly carbon intensive activity. It has not been possible to include all these environmental costs associated with the lives of those included in this study.

## Conclusions

This study confirms previous findings [8, 12, 14, 16], that services for PD can result in an overall reduction in financial costs for the wider health care system by incorporating savings from reduced subsequent health care use. This study goes further by being the first to provide a cost-effectiveness analysis of a modern, community-based approach of PD services; one which provide TC interventions across a range of intensities from 5 to 25 h per week. This model is currently more widespread compared to previous inpatient or highly intensive variants of TCs that are represented in the literature [12–15]. Furthermore, this study is the first of its kind to identify and estimate the carbon footprint of a secondary mental health service and provides some evidence for how integrating such a service can reduce the carbon footprint of the wider health care system.

The results show that TCs have the potential to reduce the overall carbon footprint of people with PD but it is likely that its impact is far wider. Preventative and empowering services such as these, which use resources efficiently to deliver high quality care for patients, are what commissioners want to invest in to ensure financial and carbon efficiency [24].

The design of new models of care in mental health requires many factors to be taken into account, including clinical effectiveness, cost effectiveness and user and carer views. We argue that the carbon footprint of clinical pathways should also be a factor that is considered when creating new models of care. However, there is no current consensuses about how this should be included, let alone measured [3, 25, 26]. The analysis provided here is a first attempt to demonstrate an approach to account for how the carbon footprint of different models of clinical care can be evaluated.

There are many constraints on the NHS [27] and budgets for health providers are being effectively cut on an annual basis [28]. Given this context, there is a lack of incentive for service providers to focus on reducing carbon footprints, despite the carbon reduction targets of the Climate Change Act. However, what this study has shown is that acting to improve patient care can potentially reduce the financial cost and carbon footprint simultaneously. Improving the sustainability of mental health care and of the wider NHS will involve identifying and adopting win-win practices, where improvements in patient’s health will also reduce both the financial cost and carbon footprint. This simple study suggests that win-win practices probably exist throughout the NHS but are not being identified.
